# Expanding energy envelope in holographic display via mutually coherent multi-directional illumination

**DOI:** 10.1038/s41598-022-10355-0

**Published:** 2022-04-22

**Authors:** Dukho Lee, Kiseung Bang, Seung-Woo Nam, Byounghyo Lee, Dongyeon Kim, Byoungho Lee

**Affiliations:** School of Electrical and Computer Engineering, Seoul National University, Gwanak-Gu Gwanakro 1, Seoul, 08826 South Korea

**Keywords:** Engineering, Optics and photonics

## Abstract

Holographic display is considered as the most promising three-dimensional (3D) display due to its unique feature of reconstructing arbitrary wavefronts. However, the limited étendue, which hinders the immersive experience of observers, remains a major unresolved issue in holographic display technique. In this paper, we propose a novel approach to tweak the constraints of étendue by expanding the energy envelope in holographic display via mutually coherent multi-illumination. The proposed concept contains both a light source design for generating a mutually coherent multi-directional wave and a computer-generated hologram optimization framework for providing high-resolution 3D holograms. To verify the proposed approach, a benchtop prototype of a holographic near-eye display providing an intrinsic large exit-pupil is implemented. The experimental results clearly show that the exit-pupil is effectively expanded by four times and an appropriate viewpoint image is reconstructed according to the view position.

## Introduction

Holographic display is considered as the most promising three-dimensional (3D) display because of the unique feature capable of reconstructing arbitrary wavefronts^1–4^. Utilizing its featural approach, this technique provides a large degree of freedom such as vision correction^5, 6^, optical aberration correction^7–9^, generation of all human visual cues^10^, and continuous depth expression^11^.

The étendue of a display is defined as the product of the solid angle and the area of the emitting light, which corresponds to the product of the field-of-view (FoV) and eyebox size in a holographic near-eye display^12–14^. Unlike conventional two-dimensional (2D) display, étendue of holographic display is limited due to its fundamental nature of light diffraction^15, 16^. The limited étendue of a holographic display is one of the bottlenecks in supporting immersive viewing conditions for users, such as wide FoV and large eyebox size^17^.

In a holographic display, a spatial light modulator (SLM) which modulates the wavefront of an incident wave is a core device, and the limited étendue issue is closely related to its pixelated structure. The factors limiting étendue resulting from the pixelated structure of the SLM can be divided into two factors. The first factor is the replication of the reconstructed signal. Because the periodic pixelated structure of the SLM generates repetitive signals called high-order terms, the bandwidth of an independent signal is limited by a width of a single-order term^18^. The second factor is the energy envelope of the reconstructed signal. The energy envelope on spatial frequency domain is determined by the Fourier transform of a single pixel^19–21^. Thus, the energy envelope is considered as a $\text {sinc}$ function, and its width is determined by the width of the active region.

As a method to overcome these two factors limiting étendue, a static scattering mask is adopted to augment the SLM^15, 16, 22, 23^. A scattering mask has a non-periodic structure or a pixelated structure smaller than pixel pitch of the SLM, and these masks serve to break the constraints of the pixelated structure of the SLM. Each of the scattering masks expands étendue by breaking the replication of the reconstructed signal or expanding the bandwidth of the reconstructed single-order term. However, the image quality is inevitably degraded since the expanded étendue is covered by the equivalent amount of information determined by the number of SLM pixels. A computer-generated hologram (CGH) optimization algorithm to enhance image quality has been proposed, but image quality is very sensitive to the errors from manufacturing process of a scattering mask and system alignment^15^.

In the current condition when the limited amount of information of the SLM is used^24, 25^, it is a more efficient way that a CGH is optimized only for a limited area and the optimized area is shifted according to the observer’s position by using tracking device. When we utilize this approach, we can tweak the constraints of étendue by expanding only the second limiting factor, the energy envelope, without considering the first factor, a bandwidth of a single-order term. The straight forward solution for expanding the energy envelope is to use multi-directional illumination. However, in holographic display, it has not been proposed to adopt multi-directional illumination because crosstalk occurs due to overlap between high-order diffracted signals from each directional wave.

In this paper, we propose a novel concept that enables multi-illumination holographic display for energy envelope expansion. The key idea of the proposed concept is to provide an additional degree of freedom to the CGH synthesis process by using a mutually coherent multi-directional wave instead of a mutually incoherent multi-directional wave. The mutual coherence between each directional wave makes it possible to control the overlap on complex amplitude basis between the reconstructed high-order terms from each directional wave. The proposed concept contains both a light source design for generating a mutually coherent multi-directional wave and a CGH optimization framework for reconstructing crosstalk-free signals. In order to increase the image quality of holographic display, various types of CGH synthesis algorithm based on optical aberration correction has been used^26–28^. The proposed CGH optimization algorithm also considers real-world artifacts such as chromatic wavelength difference, optical aberrations, and system misalignment. The proposed aberration measurement system is based on a digital holographic measurement, and the proposed approach allows the system to be tolerant from the practical conditions. The proposed CGH optimization targeting a specific region allows an effective usage of the information, physically constrained by the number of SLM pixels.

The proposed concept is verified by applying it to a holographic near-eye display. We demonstrate a benchtop prototype of a holographic near-eye display providing an intrinsic large exit-pupil, thus expanding eyebox solely with updates of CGHs. The experimental results clearly show that the energy envelope of the holographic near-eye display is widened, and thus the exit-pupil of the system is expanded.

## Results

### System implementation

**Figure 1:**
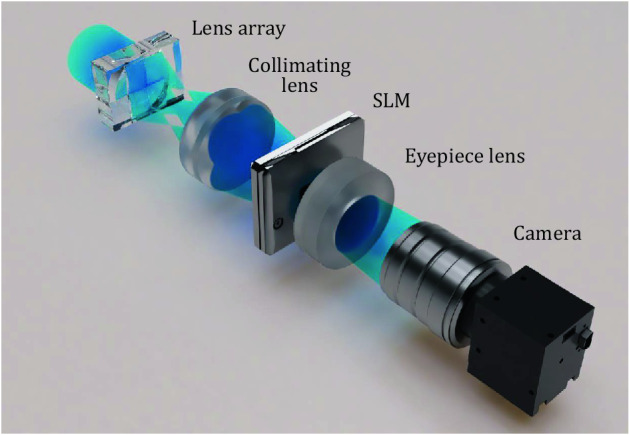
Schematic diagram of an implemented holographic near-eye display with expanded eyebox. A mutually coherent multi-directional wave generator is realized by using a lens array and a collimating lens. An eyepiece lens is placed behind the SLM to form an expanded exit-pupil.

In order to verify the proposed holographic display with expanded energy envelope, a benchtop prototype of a holographic near-eye display system for expanded eyebox is implemented as shown in Fig. 1. As a mutually coherent multi-directional wave generator, a lens array and a collimating lens are adapted in the system. A single beam from a coherent light source forms a mutually coherent point light source array by a lens array, and the formed point light sources are collimated as a mutually coherent multi-directional wave. By converting the plane wave propagating in the vertical direction of the SLM into multi-directional plane waves, the energy of the original single wave is redistributed into directional plane waves. The wavefront modulated by the SLM forms an expanded exit-pupil of the system, and the size and position of the target eyebox can be adjusted through the upload of the optimized CGH.

For the lens array and collimating lens as a mutually coherent multi-directional wave generator, a 2 × 2 lens array with a pitch of 7.5 mm and a 48 mm focal length and a collimating lens with a 105 mm focal length are used. In order to configure a full color holographic display system, we use light sources with wavelength of 457 nm, 532 nm, and 635 nm. A 1080p phase only SLM (Thorlabs, EXULUS-HD1) is used in the system.The pixel pitch and fill factor of the SLM are 6.4 μm and 93$\%$, respectively. An eyepiece lens with an 85 mm focal length is used. If we consider a conventional holographic near-eye display without multi-illumination, a field of view of approximately ${9.5}^{\circ }$ in diagonal direction and an eyebox size of 6 mm × 6 mm would be obtained. However, by using an implemented multi-illumination holographic near-eye display, the eyebox size can be expanded to 12 mm × 12 mm without compromising a field of view.

### Experimental results

#### Fourier hologram on eyebox plane

**Figure 2:**
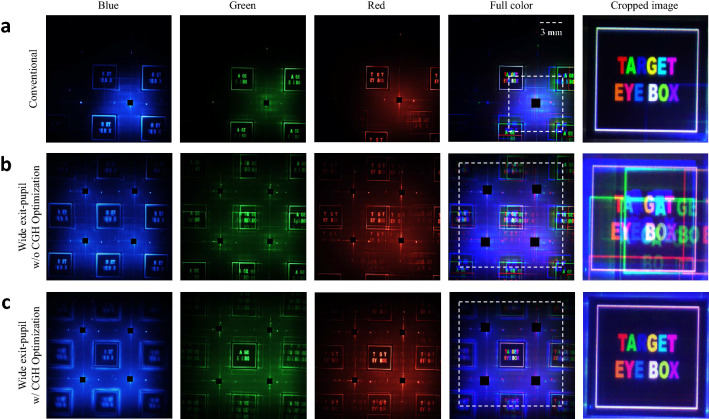
Fourier holograms reconstructed for eyebox visualization. (**a**) Reconstructed images from the conventional holographic near-eye display. Reconstructed images using proposed wide eyebox holographic near-eye display (**b**) with a conventional non-optimized CGH and (**c**) with an optimized CGH. The position and size of the region of interest are set to the center of the expanded exit-pupil and 3 mm × 3 mm, respectively.

In the implemented holographic near-eye display system, the eyebox plane is placed on the Fourier domain of the SLM. In order to visualize the eyebox of the system, Fourier hologram is reconstructed as shown in Fig. 2. Figure 2a represents the reconstructed images from the conventional holographic near-eye display, and Fig. 2b,c show the reconstructed images without and with CGH optimization in the proposed wide eyebox holographic near-eye display, respectively. Each red, green, and blue color image is obtained by digitally combining 15 different random phase images for speckle reduction. Full color image is synthesized by combining the image for each color without any image process. The DC noises are digitally blocked.

**Figure 3:**
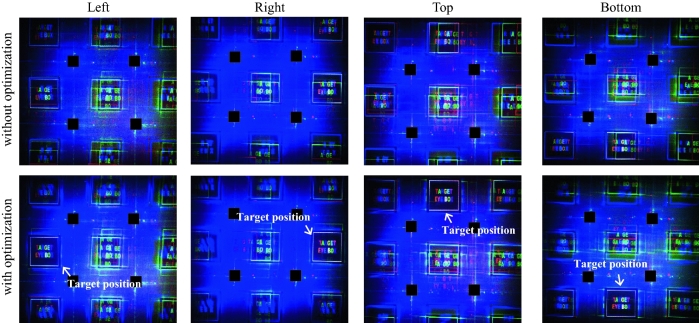
Reconstructed Fourier holograms from the non-optimized and optimized CGH for the left, right, top, and bottom position on the eyebox plane.

The exit-pupil size of the system is indicated by a white grid on the full color image. Conventional holographic near-eye display provides exit-pupil of 6 mm × 6 mm size, same as bandwidth size of the SLM, but when using the proposed system, it is confirmed that it provides exit-pupil of 12 mm × 12 mm size, which is 4 times larger than the conventional size. Note that the size of the bandwidth of the SLM is calculated based on the blue light source with a wavelength of 457 nm. The size of the target eyebox in the exit-pupil is set to 3 mm × 3 mm, which is 0.5 times the size of the SLM bandwidth, and the corresponding region of interest is designated in the CGH optimization algorithm. The region of interest on Fourier domain is cropped and shown in the last column of Fig. 2. Experimental results definitely show that the optimized CGH effectively eliminates crosstalk and reconstructs clear target images. Figure 3 shows the reconstructed images from the non-optimized and optimized CGH for the left, right, top and bottom position, in addition to the center position of the exit-pupil shown in Fig. 2. The results show that the position of the target eyebox can be effectively shifted by only updating the optimized CGH where the position of the region of interest is shifted. This indicates that it can provide an expanded eyebox when combined with a tracking device.

#### 3D image display result

**Figure 4:**
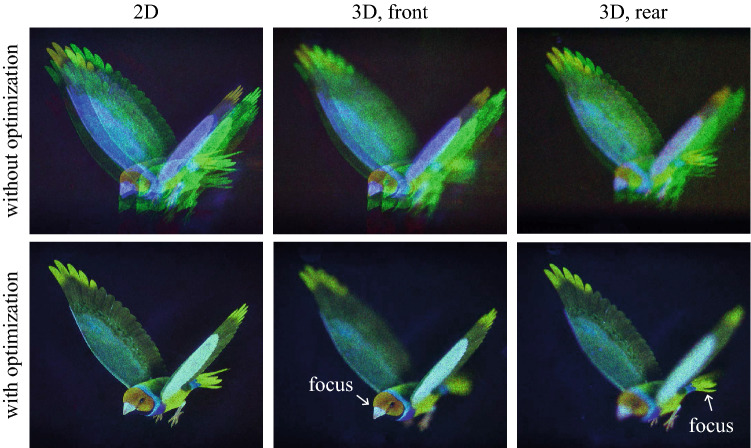
Full color finch images observed from the center viewpoint of the exit-pupil. A 2D finch image and 3D images with focus on the front and rear are reconstructed with and without CGH optimization. The finch image is purchased from TurboSquid, All Rights Reserved.

**Figure 5:**
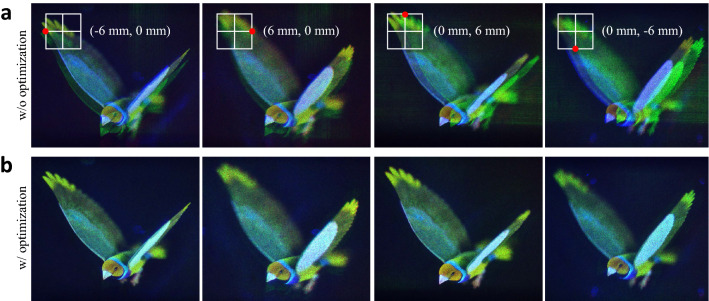
Viewpoint images observed at a position shifted by 6 mm to the left, right, top, and bottom with respect to the center position within the expanded exit-pupil. (**a**) A non-optimized CGH generates crosstalk, whereas (**b**) an optimized CGH reconstructs a target finch image without crosstalk. The finch image is purchased from TurboSquid, All Rights Reserved.

Next, Fresnel holograms for the target viewpoint image are reconstructed. Figure 4 shows full color finch images observed from the center viewpoint of the exit-pupil. A 2D finch image and 3D images with focus on the front and rear are shown. Each finch image is digitally synthesized using individually captured red, green, and blue images like Fourier hologram results, and each red, green, and blue image is obtained by a superposition of 15 different random phase images for speckle suppression. The 2D finch image is reconstructed 62 mm from the SLM, and the 3D finch image has a height of 12.3 mm, a width of 6.9 mm and a depth of 62 mm. The center of the 3D finch is placed 93 mm from the SLM. When the conventional non-optimized CGH is reconstructed by using the proposed system, crosstalk occurs, but when the optimized CGH is used, the crosstalk is effectively removed as shown in Fig. 4. As shown in Fig. 5, the CGH for the shifted region of interest provides an appropriate viewpoint image. Each viewpoint image is observed at a position shifted by 6 mm to the left, right, top, and bottom with respect to the central position within the expanded exit-pupil. Each viewpoint image is focused on the front of the finch. The experimental result clearly shows that the reconstructed image from the non-optimized CGH contains crosstalk, but only the target image is reconstructed when the optimized CGH is used.

### Simulation results

The simulation is conducted to verify the validity of the proposed concept and to analyze the major factors that critically affect crosstalk of a reconstructed signal. We simulate the proposed concept of expanding energy envelope in holographic display by using Python running on an Nvidia GeForce RTX 2060 GPU. In order to focus on the factors that affect crosstalk, we consider only two directional waves constituting a multi-directional wave. An SLM with 6.4 μm pixel pitch, 1920 × 1080 resolution, and 93$\%$ fill factor is used. Wavelength of the reference wave is 532 nm, thus the maximum diffraction angle is calculated to $\pm {2.38}^{\circ }$. Note that the maximum diffraction angle is limited within the 0th order term of the reconstructed signal. We assume that one of the two directional waves is incident normal to the SLM, and the other is incident at an angle of ${2.04}^{\circ }$. The region of interest is defined on the Fourier plane of the SLM. The position is set at the mid-position of the two directional waves, and the size is set to 0.5 times the bandwidth of the SLM.

**Figure 6:**
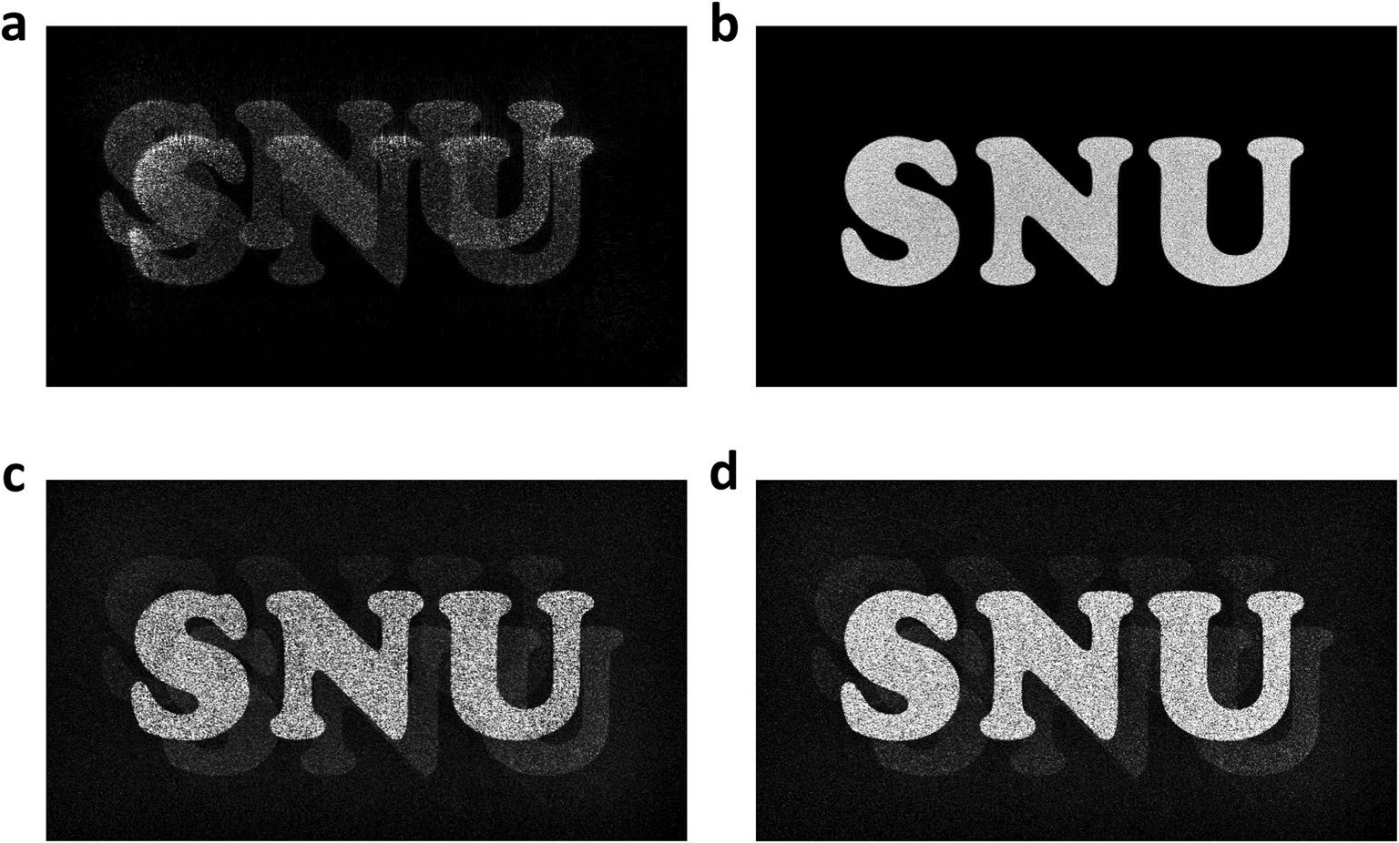
Simulation results considering two directional waves. Reconstructed images from (**a**) a conventional non-optimized CGH and (**b**) an optimized CGH. The simulation result of reconstructing the optimized CGH (**c**) with one plane wave and (**d**) the other plane wave.

Optical aberrations for two directional plane waves are also taken into account. In the simulation, it is assumed that the optical aberration to be experimentally measured is known. The phase map representing optical aberration is used for both CGH optimization and CGH reconstruction process. The reconstructed images from the conventional non-optimized CGH and the optimized CGH are shown in Fig. 6a,b. The conventional non-optimized CGH is calculated by wave propagation theory based on angular spectrum method^29^ and only considering a wave which is incident normal to the SLM. When the conventional CGH is used, crosstalk appears as shown in Fig. 6a due to misalignment and aberration of the reconstructed signals. When the optimized CGH is reconstructed, the crosstalk is eliminated and the target image is reconstructed well as shown in Fig. 6b. Figure 6c,d show the results of reconstructing the optimized CGH with only one of the two directional waves. It is shown that the reconstructed images from each directional wave are aligned with each other, and the target signal and crosstalk signal are intensified and removed, respectively.

**Figure 7:**
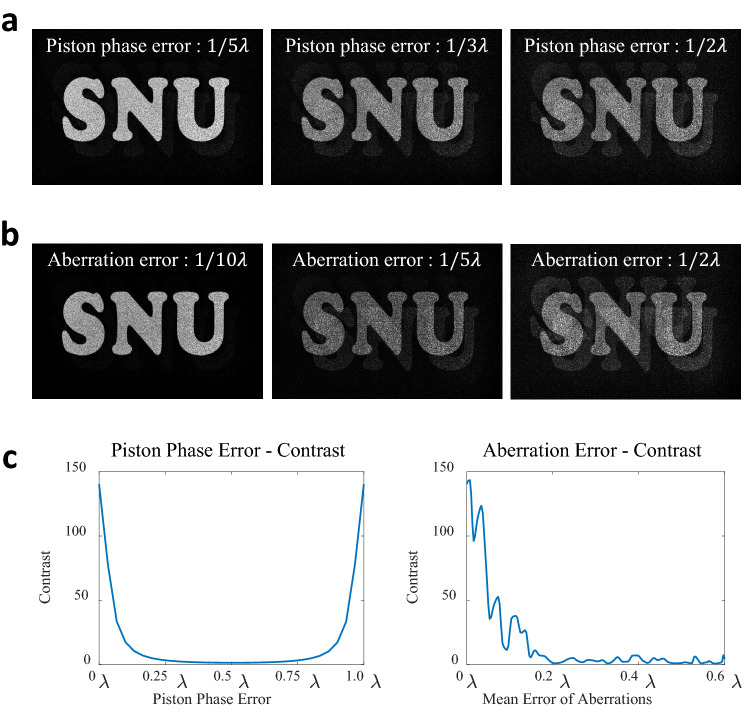
Simulation results for analyzing the factors affecting crosstalk. The reconstructed images with crosstalk according to (**a**) the piston error and (**b**) the aberration error. (**c**) Weber contrast versus the piston error and the aberration error.

In order to test the sensitivity of the unexpected error, we simulate the reconstruction of the optimized CGH under the condition that the unexpected error exists. We divided the major factors affecting crosstalk into two categories: the piston phase error and the aberration error. The piston phase error represents the difference between the ground truth piston phase and measured piston phase of two directional plane waves. One of the two directional plane wave is taken as the reference for the piston phase. When a piston phase of the reference directional plane wave is considered as zero, the piston phase error is determined to the difference between the ground truth piston phase of another plane wave and the measured piston phase of the plane wave. The aberration error represents the root mean square error over the phase difference between the ground truth optical aberration and the measured aberration. Figure 7a,b show the reconstructed images including crosstalk according to the piston phase error and the aberration error, respectively. Both results represent that the larger the error, the more severe the crosstalk. We evaluate the crosstalk of the reconstructed image for the piston phase error and the aberration error by calculating Weber contrast for the intensity of the target signal and the intensity of the ghost signal caused by incomplete destructive interference. The target signal means a signal that should be intensified by constructive interference, and the ghost signal means a signal that should be eliminated by destructive interference but remains due to errors. Figure 7c is plots representing Weber contrast for the piston phase error and the aberration error. It shows that the crosstalk is sensitive to both errors, and it is confirmed that both errors should be smaller than 1/20 wavelength in order to obtain a Weber contrast value of about 120 or more. The simulation results show that the piston phase error and the aberration error sensitively affect the crosstalk, and these two factors should be accurately measured.

### Interferometer for measuring the optical aberration

**Figure 8:**
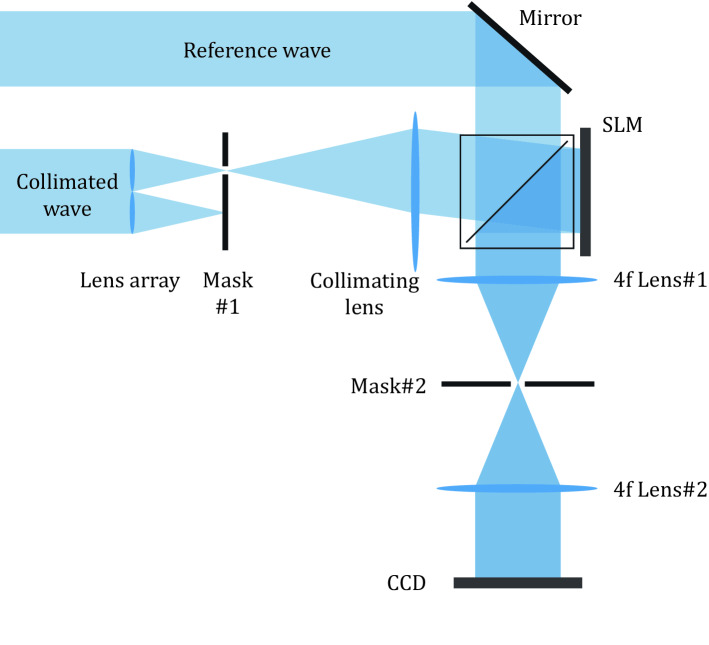
Schematic diagram of a Mach-Zehnder interferometer setup for wavefront measurement of a mutually coherent multi-directional wave. The spatial mask #1 is opened corresponding to the lenslet whose optical aberration is to be measured, and the mask #2 selects the position on the spatial frequency domain where optical aberration is to be measured.

A Mach-Zehnder interferometer is configured to measure and pre-compensate the optical aberration for each lenslet, as shown in Fig. 8. We measure the aberrations on the SLM plane by using an interference pattern between a reference wave and each directional wave constituting the multi-directional wave. In order to obtain each aberration at the target spatial frequency, the interference pattern is measured after each directional wave is sequentially selected and the reference wave is rotated to the target spatial frequency coordinate.

The first spatial mask is placed on the focal plane of the lens array, and the mask is opened corresponding to the lenslet whose optical aberration is to be measured. The optical aberrations of the proposed system are under spatially varying conditions, so we must measure the aberration separately according to the position of the region of interest on the eyebox plane. A second spatial mask is introduced into the 4f system of the SLM to select the position on the eyebox plane where optical aberrations are to be measured. After the lenslet and the target position on the eyebox plane to be measured are set, the interference pattern between the reference wave and the directional plane wave generated by the lenslet is measured. Since the CGH optimization algorithm is designed to take into account the optical aberration of the directional wave on the SLM plane, the interference pattern is also measured on the SLM plane. The phase map displayed on the SLM is generated based on the Legendre polynomials. In order to obtain a phase map that compensates for the optical aberration of the target plane wave, we find Legendre coefficients that flatten the interference pattern through precise adjustment of the coefficients. The flat interference pattern means that the wavefront modulated by the SLM is equal to the reference wave within the measurement error. The optical aberration of the target lenslet at the target position on the eyebox can be determined using the phase map displayed on the SLM. After measuring the optical aberration of all lenslets composing the lens array at target position of eyebox plane, the optical aberrations are applied to the optimization algorithm to obtain a phase-only CGH considering aberrations.

**Figure 9:**
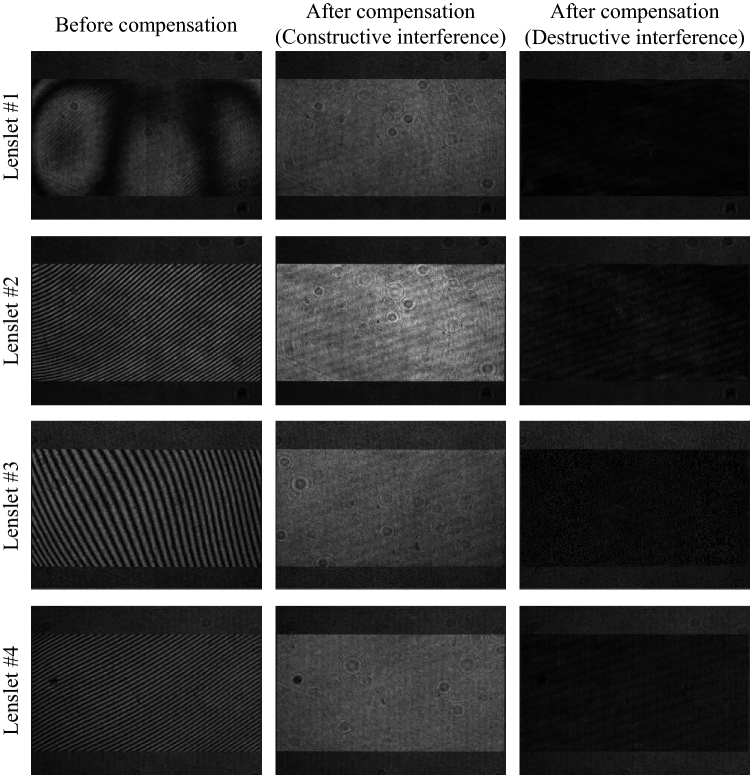
The interference pattern between the reference plane wave and the directional plane wave generated by lenslet #1 to lenslet #4. The aberration pattern is flattened by compensation using the measured wavefront. By changing the piston phase of the wavefront, constructive and destructive interference can be controlled.

Because the optical aberration changes with spatial frequency, it is practically impossible to measure the entire aberration of the system. Therefore, the aberrations at the center position of the region of interest are measured and used as an approximation. We measure the aberrations for five target positions of the center, right, left, top, and bottom of the expanded exit-pupil. Also, the aberrations for each position are measured for the full color light sources. Among the measured aberrations, the result for the green light source at the center position is shown as an example in Fig. 9. The interference patterns in Fig. 9 show that a proper phase map that compensates for optical aberrations is found, and constructive and destructive interference can be controlled by changing the piston phase of the phase map. The aberration error of the measured phase map is evaluated by the Strehl ratio. All measured phase maps have a Strehl ratio higher than 0.97, which means that the root mean square error over the phase difference is less than 1/40 wavelength.

The piston phase of each directional plane wave is found after setting one plane wave as the reference for the piston phase. In order to find the piston phase of each plane wave, the experiment is conducted after opening only two lenslets, a reference lenslet and a target lenslet. Since the lens array implemented in the system consists of 2 × 2 lenslets, a piston phase acquisition experiment is performed for a total of 3 lenslet pairs. As with the optical aberration measurement process, it is conducted for each of the five positions and full color light sources. In order to find the piston phase of the target plane wave, CGHs are synthesized while scanning the piston phase. After capturing the reconstructed image from the synthesized CGHs, we select the piston phase with the highest Weber contrast value.

When the optical aberration and the piston phase are measured for all lenslets constituting the lens array, full color CGHs for each target position can be optimized by considering the mutually coherent multi-directional wave generated by the entire 2 × 2 lenslets.

## Discussion

We have demonstrated a novel approach to overcome the limited étendue issue by expanding the energy envelope in holographic display via mutually coherent multi-directional illumination. By using the proposed approach, the energy envelop in holographic display is intrinsically expanded and the crosstalk-free, high-resolution 3D hologram can be reconstructed at any position within the expanded energy envelope solely with updates of CGHs. To verify the proposed optical design and CGH optimization framework, a benchtop prototype of full color holographic near-eye display is implemented. The simulation and experimental results clearly show that the full color crosstalk-free 3D images are observed within the intrinsically expanded exit-pupil.

Until now, a multi-illumination holographic display has not been developed due to its crosstalk from the overlap between reconstructed high-order terms from each directional wave. The proposed method is the first approach to enable multi-illumination holographic display by eliminating crosstalk via precise control of interference between signals reconstructed from each directional wave. Under the condition when the limited amount of information of the SLM is used, it would be an efficient approach to expand a limited eyebox via the proposed approach after designing a holographic near-eye display system with wide field of view. We hope that the proposed concept can provide a novel solution for implementing a practical and stable holographic near-eye display since the optical system does not contain any active optical components.

## Methods

### Expanding energy envelope in holographic display via mutually coherent multi-directional illumination

**Figure 10:**
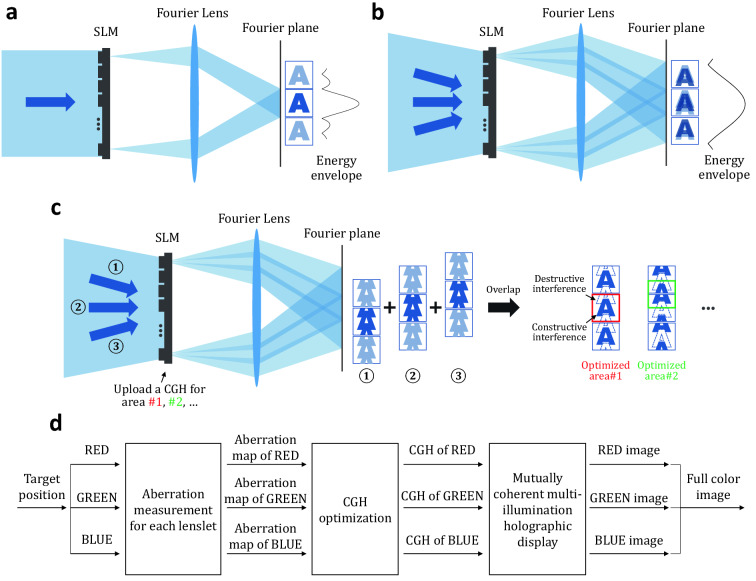
A schematic diagram of multi-illumination holographic displays. (**a**) A conventional single-illumination holographic display generates the narrow energy envelope. (**b**) A multi-illumination adopting to a holographic display can effectively expand the energy envelope, but the reconstructed signals should include critical crosstalk. (**c**) In order to generate crosstalk-free signals, we adopt a mutually coherent multi-illumination to a holographic display. The mutual coherence between each directional wave makes it possible to intensify a target signal and cancel crosstalk signal by constructive and destructive interference, respectively. The optimized area where the target signal is reconstructed can be shifted solely with updates of CGHs. (**d**) A flow chart of the proposed experimental process including optical aberration measurement, CGH optimization, and multi-illumination holographic display.

Figure 10 represents a schematic diagram of a conventional holographic display and the proposed multi-illumination holographic display. A conventional single-illumination holographic display reconstructs signals in the narrow energy envelope as shown in Fig. 10a. When we adopt a multi-directional illumination to a holographic display, the energy envelope is effectively expanded, but the reconstructed signal contains critical crosstalk as shown in Fig. 10b. Each of the directional waves generates repetitive high-order terms, and the overlap between the repetitive signals is the main cause of crosstalk. Assuming an ideal optical system without any artifacts such as optical aberrations and system misalignment, crosstalk-free signals could be reconstructed by overlapping between perfectly aligned high-order signals diffracted from each directional wave. However, it is an overstrict approach to design and implement a perfect aberration-free optical system in very precise alignment for all directional waves.

Figure 10c represents the proposed concept of reconstructing crosstalk-free signals by using a mutually coherent multi-directional wave instead of a mutually incoherent multi-directional wave. The mutually coherent multi-directional wave is generated by a single coherent light source rather than multiple directional coherent light sources. When we adopt a mutually coherent multi-directional wave, the reconstructed signals from each directional wave are added on complex amplitude basis, so crosstalk can be canceled by destructive interference. As shown in Fig. 10c, each reconstructed signal corresponding to the directional wave includes both the target signal and an additional pre-compensation signal. The target signal is intensified and crosstalk signal is eliminated by constructive and destructive interference, respectively. The flow chart of the proposed experimental process is shown in Fig. 10d. After setting the target pupil position in the eyebox domain, the optical aberration at the target position for each lenslet composing a lens array is measured. Based on the measured optical aberration maps, the CGHs are optimized. The optimized CGHs are reconstructed by using the proposed multi-illumination holographic display and the reconstructed images are captured. All processes are performed separately for red, green, and blue color, and finally a full color image is obtained.

In order to realize the proposed concept, it is essential to precisely control both the alignment and phase difference between the signals to interfere. We utilize a CGH optimization framework based on digital holographic wavefront measurement of each directional wave. By using the proposed CGH synthesis method, we can precisely control both the alignment and phase difference with an accuracy of wavelength scale. Also, the misalignment accordance with the wavelength of light source can be considered. The position of the crosstalk-free reconstructed signal is changed according to the position of the region of interest in the optimization process, and it allows to reconstruct the desired signal within the expanded energy envelope solely by uploading the optimized CGH. The detailed algorithm is discussed in the next section.

### Computer-generated hologram synthesis algorithm

Theoretically, assuming an ideal SLM is used, it is possible to pre-compensate the wavefront of the mutually coherent multi-directional wave by dividing the target CGH pattern to the wavefront of the reference wave. However, commercially available SLMs can only modulate the phase or amplitude of the incident wave, and the bandwidth of the expressible signal is limited due to the resolution of the SLM. Thus, the reference wave cannot be directly compensated by the SLM pattern.

We propose a CGH optimization algorithm, and the region of interest is set within the bandwidth of the SLM on the spatial frequency domain. The center frequency of the region of interest can be shifted to any position within the expanded energy envelope. In order to optimize a valid CGH pattern ${{\hat{\phi }}_{\text {SLM}}}$ for the system, we set an error minimization problem. The problem is set to minimize the loss between the reconstructed wavefront from the CGH pattern and the target complex amplitude. The proposed error minimization problem is as follows.

1 $$\begin{aligned} {{\hat{\phi }}_{\text {SLM}}}=\underset{{\hat{\phi }}}{\mathop {\text {arg min}}}\,\,\,\,\,\mathscr {L}\left( f\left( {\hat{\phi }} \right) ,\,{{{\hat{h}}}_{\text {target}}};\,\hat{M} \right) , \end{aligned}$$

where $\hat{\phi }$ is the phase profile of the SLM, ${{\hat{h}}_{\text {target}}}$ is a target complex amplitude hologram calculated from the target image, $\hat{M}$ is a binary mask for designating region of interest, the function $f\left( \cdot \right)$ is forward propagation function that outputs a complex amplitude wavefront, and $\mathscr {L}\left( \cdot ,\,\cdot \right)$ represents a loss function between two complex amplitude matrices. Note that the optimization problem is for phase-only CGH since the system adopts phase-only SLM. We use Adam optimization algorithm, and our CGH optimization algorithm is summarized in Algorithm 1. 
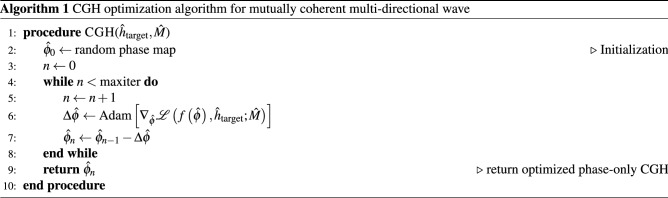


Although Eq. (1) is represented as a discrete matrix form, the following equations are described as continuous form for an intuitive expression. The relation between the two forms are as follows.

2 $$\begin{aligned} \begin{array}{*{35}{l}} \hat{\phi }\left[ m,n \right] =\phi \left( dx\cdot m,dy\cdot n \right) , \\ {{{\hat{h}}}_{\text {target}}}\left[ m,n \right] ={{h}_{\text {target}}}\left( dx\cdot m,dy\cdot n \right) , \\ \end{array} \end{aligned}$$

where $\phi \left( \cdot ,\,\cdot \right)$ and ${{h}_{\text {target}}}\left( \cdot ,\,\cdot \right)$ are the phase-only hologram and the target complex amplitude hologram, respectively, defined on continuous domain, *m* and *n* are column and row indices of the matrix, respectively, and *dx* and *dy* are pixel pitches of the SLM corresponding to the *x*-axis and *y*-axis, respectively.

The loss function is defined for solving complex-valued optimization problems on region of interest of the spatial frequency domain. We use the loss function based on two complex amplitude wavefront maps instead of intensity maps to reduce computational complexity. Since we adopt loss function for complex target, all of the depth information can be considered in a single plane. Thus, the number of depths of the target 3D contents does not significantly affect the computational load. Also, the binary mask *M* is adopted to designate a region of interest on spatial frequency plane. The binary mask *M* sets the region of interest to 1 and the non-interested region to 0. The position and bandwidth of the region of interest can be defined within the expanded energy envelope. The loss function and the binary mask is as follows.

3 $$\begin{aligned} \begin{array}{*{35}{l}} \mathscr {L}\left( f\left( \phi \right) ,{{\text {h}}_{\text {target}}};M \right) ={{\left\| \text {iFT}\left[ M\cdot \text {FT}\left[ f\left( \phi \right) -{{\text {h}}_{\text {target}}} \right] \right] \right\| }^{2}}, \\ \\ M\left( {{f}_{x}},{{f}_{y}} \right) =\left\{ \begin{array}{*{35}{l}} \,\,1\,,\,\,\,\text {if}\,\sqrt{{{\left( {{f}_{x}}-{{f}_{RoI,x}} \right) }^{2}}+{{\left( {{f}_{y}}-{{f}_{RoI,y}} \right) }^{2}}}<\rho \\ \,\,0\,,\,\text {otherwise} \\ \end{array}, \right. \, \\ \end{array}\ \end{aligned}$$

where $\text {FT}\left[ \cdot \right]$ and $\text {iFT}\left[ \cdot \right]$ denote a 2D Fourier transform and an inverse 2D Fourier transform, respectively, ${{\left\| \cdot \right\| }^{2}}$ denotes an integration of absolute square of a complex-valued function, *M* represents the binary value accordance with the position of $\left( {{f}_{x}},\,{{f}_{y}} \right)$ on the spatial frequency domain, ${{f}_{RoI,x}}$ and ${{f}_{RoI,y}}$ is the center spatial frequencies of the region of interest corresponding to the ${{f}_{x}}$-axis and ${{f}_{y}}$-axis, respectively, and $\rho$ represents radius of region of interest.

The forward propagation function $f(\cdot )$ describes an optical process in which the CGH pattern is modulated by a mutually coherent multi-directional wave. In forward propagation function, the generated reference wave is considered as an integration of multi-frequency plane waves. The repetitive signals generated by each plane wave are calculated separately and overlapped on each other. Each modulated wave is overlapped on Fourier domain of the SLM and then transformed into the spatial domain of the SLM. Optical aberrations for each plane wave, including system misalignment, are also considered. As the optical aberrations, the wavefront obtained by digital holographic measurement is used. The mathematical model of the forward propagation function is described as follows.

4 $$\begin{aligned} f\left( \phi \right) =\text {iFT}\left[ \sum \limits _{k\le {{N}_{m}}}{{{E}_{k}}\left( {{f}_{x}},{{f}_{y}} \right) \cdot \text {FT}\left[ \exp \left( j\phi \left( x,y \right) \right) \cdot {{d}_{k}}(x,y)\cdot r\left( x,y \right) \right] } \right] ,\ \end{aligned}$$

where ${{N}_{m}}$ is the number of directional waves composing the reference wave, *k* is an index of the directional wave, ${{E}_{k}}\left( \cdot ,\,\cdot \right)$ is shifted $\text {sinc}$ envelope corresponding to the *k*-th directional wave, considering the region of interest, directional wave, and the aberration, ${{d}_{k}}\left( \cdot ,\,\cdot \right)$ represents a directional wave considering its aberrations, and $r\left( \cdot ,\,\cdot \right)$ is a grating that shifts the reconstructed signal from the origin to the center position of region of interest. ${{d}_{k}}\left( \cdot ,\,\cdot \right)$ and $r\left( \cdot ,\,\cdot \right)$ act as a circular shift operator by Fourier transform for carrier frequencies beyond the bandwidth of the SLM. The detailed expression for ${{E}_{k}}\left( \cdot ,\,\cdot \right)$, ${{d}_{k}}\left( \cdot ,\,\cdot \right)$, and $r\left( \cdot ,\,\cdot \right)$ is as follows.

5 $$\begin{aligned} \begin{array}{*{70}{l}} {{\mathrm{{E}}_k}\left( {{f_x},{f_y}} \right) = \left[ {\mathrm{{sinc}}\left[ {{\alpha _x}\left( {{f_x} + {f_{RoI,x}} - {f_{k,x}}} \right) } \right] \mathrm{{sinc}}\left[ {{\alpha _y}\left( {{f_y} + {f_{RoI,y}} - {f_{k,y}}} \right) } \right] } \right] }{ \otimes \,\mathrm{{FT}}\left[ {{a_{k,{f_{RoI,x}},{f_{RoI,y}}}}\left( {x,y} \right) } \right] },\\ {{d_k}\left( {x,y} \right) = \exp \left[ {j2\pi \left( {{f_{k,x}}x + {f_{k,y}}y} \right) } \right] \cdot {a_{k,{f_{RoI,x}},{f_{RoI,y}}}}\left( {x,y} \right) },\\ {r\left( {x,y} \right) = \exp \left[ { - j2\pi \left( {{f_{RoI,x}}x + {f_{RoI,y}}y} \right) } \right] ,} \end{array} \end{aligned}$$

where ${{\alpha }_{x}}$ and ${{\alpha }_{y}}$ are widths of active area of the SLM corresponding to the *x*-axis and *y*-axis, respectively, ${{f}_{k,x}}$ and ${{f}_{k,y}}$ are spatial frequencies of the *k*-th directional wave, the symbol $\otimes$ denotes convolution operator, and ${{a}_{k,{{f}_{RoI,x}},{{f}_{RoI,y}}}}\left( \cdot ,\,\cdot \right)$ is the aberration of *k*-th directional wave at the position of $\left( {{f}_{RoI,x}},{{f}_{RoI,y}} \right)$ on the spatial frequency domain. The measured aberration ${{a}_{k,{{f}_{RoI,x}},{{f}_{RoI,y}}}}\left( \cdot ,\,\cdot \right)$ is based on Legendre polynomials, and it is expressed as follows.

6 $$\begin{aligned} {a_{k,{f_{RoI,x}},{f_{RoI,y}}}}\left( {x,y} \right) = \exp \left[ {j\sum \limits _q {{{\left. {{c_q}} \right| }_{k,{f_{RoI,x}},{f_{RoI,y}}}}{p_q}\left( {x,y} \right) } } \right] , \end{aligned}$$

where ${{c}_{q}}$ and ${{p}_{q}}\left( \cdot ,\,\cdot \right)$ stand for the *q*-th Legendre coefficients and Legendre basis function, respectively. Under the condition that optical aberrations are spatially varying, the optical aberration of each plane wave depends on the spatial frequency. It is computationally too expensive to obtain aberrations for all spatial frequencies for all plane waves. Therefore, under the assumption that the aberration is spatially slow-varying, optical aberrations are measured only for the sampled position within the extended energy distribution, and the same aberration map is used for the nearby region.

The target complex hologram ${{\hat{h}}_{\text {target}}}$ can be calculated by any conventional CGH synthesis method, such as a depth map method^30, 31^, a point cloud method^32–34^, or a polygon-based method^35, 36^. Because the target CGH reflects complex wavefront, arbitrary phase profile of the 3D image can also be considered. In this paper, we adopt the depth map CGH synthesis method based on angular spectrum propagation theorem.
